# Direct correlation of MR-DWI and histopathology of Wilms’ tumours through a patient-specific 3D-printed cutting guide

**DOI:** 10.1007/s00330-024-10959-2

**Published:** 2024-08-08

**Authors:** Justine N. van der Beek, Matthijs Fitski, Ronald R. de Krijger, Marijn A. Vermeulen, Peter G. J. Nikkels, Arie Maat, Myrthe A. D. Buser, Marc H. W. A. Wijnen, Jeroen Hendrikse, Marry M. van den Heuvel-Eibrink, Alida F. W. van der Steeg, Annemieke S. Littooij

**Affiliations:** 1 Princess Máxima Center for Pediatric Oncology, Utrecht, The Netherlands; 2 Department of Radiology and Nuclear Medicine, University Medical Center Utrecht/Wilhelmina Children’s Hospital, Utrecht, The Netherlands; 3 Department of Pathology, University Medical Center Utrecht, Utrecht, The Netherlands; 4 Division of Child Health, Wilhelmina Children’s Hospital, Utrecht University, Utrecht, The Netherlands

**Keywords:** Wilms tumour, Diffusion magnetic resonance imaging, Pathology, Paediatrics, Three-dimensional printing

## Abstract

**Objectives:**

The International Society of Paediatric Oncology-Renal Tumour Study Group (SIOP-RTSG) discourages invasive procedures to determine the histology of paediatric renal neoplasms at diagnosis. Therefore, the histological subtype of Wilms’ tumours (WT) is unknown at the start of neoadjuvant chemotherapy. MR-DWI shows potential value as a non-invasive biomarker through apparent diffusion coefficients (ADCs). This study aimed to describe MR characteristics and ADC values of paediatric renal tumours to differentiate subtypes.

**Materials and methods:**

Children with a renal tumour undergoing surgery within the SIOP-RTSG 2016-UMBRELLA protocol were prospectively included between May 2021 and 2023. In the case of a total nephrectomy, a patient-specific cutting guide based on the neoadjuvant MR was 3D-printed, allowing a correlation between imaging and histopathology. Whole-tumour volumes and ADC values were statistically compared with the Mann-Whitney *U*-test. Direct correlation on the microscopic slide level was analysed through mixed model analysis.

**Results:**

Fifty-nine lesions of 54 patients (58% male, median age 3.0 years (range 0–17.7 years)) were included. Forty-four lesions involved a WT. Stromal type WT showed the lowest median decrease in volume after neoadjuvant chemotherapy (48.1 cm^3^, range 561.5–(+)332.7 cm^3^, *p* = 0.035). On a microscopic slide level (*n* = 240 slides) after direct correlation through the cutting guide, stromal areas showed a significantly higher median ADC value compared to epithelial and blastemal foci (*p* < 0.001). With a cut-off value of 1.195 * 10^−3^ mm^2^/s, sensitivity, and specificity were 95.2% (95% confidence interval 87.6–98.4%) and 90.5% (95% confidence interval 68.2–98.3%), respectively.

**Conclusion:**

Correlation between histopathology and MR-DWI through a patient-specific 3D-printed cutting guide resulted in significant discrimination of stromal type WT from epithelial and blastemal subtypes.

**Clinical relevance statement:**

Stromal Wilms’ tumours could be discriminated from epithelial- and blastemal lesions based on high apparent diffusion coefficient values and limited decrease in volume after neoadjuvant chemotherapy. This may aid in future decision-making, especially concerning discrimination between low- and high-risk neoplasms.

**Key Points:**

- *MR-DWI shows potential value as a non-invasive biomarker in paediatric renal tumours.*
- *The patient-specific cutting guide leads to a correlation between apparent diffusion coefficient values and Wilms’ tumour subtype.*
- *Stromal areas could be discriminated from epithelial and blastemal foci in Wilms’ tumours based on apparent diffusion coefficient values.*

## Introduction

Renal tumours account for 5–7% of all paediatric malignancies; 85% of which are nephroblastoma (Wilms’ tumour, WT) [1–6]. The latter is a heterogeneous mass that develops from embryonic renal cells, containing various amounts of stromal, epithelial and blastemal components. This results in different histological WT subtypes, with varying aggressiveness [3, 7, 8]. Nephrogenic rests (NR), foci of persistent embryonal metanephric blastema, often occur in conjunction with or as a pre-stage of WT [9–12].

Regardless of these different potential diagnoses of renal malignancies, within the International Society of Paediatric Oncology-Renal Tumour Study Group (SIOP-RTSG) protocols, in case of radiologically suspected WT neoadjuvant chemotherapy is started without histological confirmation or differentiation of histological variant [13, 14]. If a different renal neoplastic entity is suspected, a biopsy might be indicated [8, 15]. This is in contrast to The Children’s Oncology Group (COG, North-America), who instead advocate upfront surgery in localised disease [16]. Nonetheless, a biopsy might not be representative of whole-tumour histology in the case of triphasic lesions. Both approaches show equal survival rates, however, WT-based chemotherapy can potentially be initiated in other suspected tumours following the SIOP-RTSG approach. The exception is when clinical and imaging characteristics raise suspicion of a non-WT, in which case a biopsy might be indicated [3, 13, 14]. Accordingly, imaging may play an increasingly fundamental role in the non-invasive discrimination of WT subtypes as well as in the differentiation of non-WTs from WT, and therefore in guiding the decision for neoadjuvant treatment and a potential biopsy [17].

MR is the standard of care within the SIOP-RTSG 2016-UMBRELLA protocol. It provides high soft tissue contrast without ionising radiation and functional tools such as diffusion-weighted imaging (DWI). Previous studies have already suggested differences in apparent diffusion coefficient (ADC) values among WT subtypes [18–24]. Nonetheless, these studies focused on the correlation of histopathology and whole-tumour ADC values, or visual comparison of slices after freehand slicing of the specimen [18, 20, 22, 23, 25]. This might obscure underlying specific correlations of histopathological subtypes. Therefore, non-invasive discrimination based on MR could be improved by directly comparing in-vivo imaging features with ex-vivo histopathological findings [21, 26, 27].

In a recent feasibility study, we reported on the workflow for a patient-specific 3D-printed cutting guide, providing a direct comparison of ADC and histopathological features of paediatric renal neoplasms [27]. The current prospective national study correlates MR findings including ADC values directly with histopathological WT subtypes, with the ultimate aim to identify MR-DWI features to discriminate WT subtypes at diagnosis.

## Materials and methods

### Patients

**Fig. 1:**
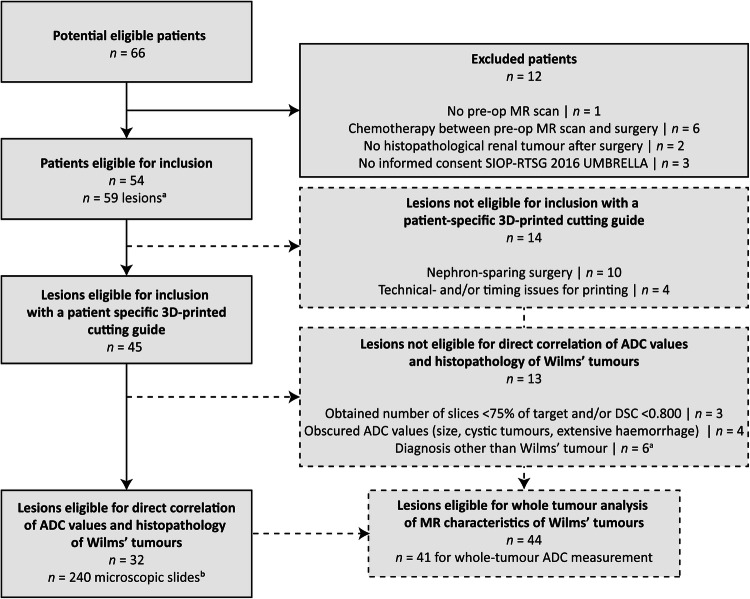
Flow diagram of patients included in the study. **a** Of the 54 included patients, three patients presented with two different lesions, whereas two patients underwent separate surgeries which were individually included, resulting in 59 lesions. To avoid ambiguity, hereafter numbers represent lesions; **b** Thirty-two lesions were found eligible for direct correlation of ADC values and histopathology after slicing with the cutting guide, resulting in a total of 240 included microscopic ‘mega’ slides, which were separately assessed concerning percentage of WT components, and correlated to the ADC value of their region of interest on the diffusion-weighted imaging-scan. MR, magnetic resonance imaging; ADC, apparent diffusion coefficient; DSC, dice similarity coefficient. ──── Patients potentially eligible for inclusion with the 3D-printed cutting guide. ─ ─ ─ Patients (potentially) eligible for whole-tumour analysis

The Institutional Review Board of the University Medical Centre Utrecht approved this prospective study and waived the requirement for a separate informed consent since research purposes and MR scans as standard of care were embedded in the UMBRELLA protocol with no additional burden for the patient following human rights declarations and regulations [27]. Between May 2021 and May 2023, 66 paediatric patients with renal tumour were considered for prospective inclusion in the study (Fig. 1). This national study was conducted in a single centre, given centralised care for paediatric oncology patients. Inclusion criteria for whole-tumour analysis were children who underwent an MR examination at diagnosis and, in case of neoadjuvant chemotherapy following the UMBRELLA protocol, a pre-operative MR scan before total nephrectomy or nephron-sparing surgery after neoadjuvant chemotherapy consisting of 4 weeks of vincristine and actinomycin-D or 6 weeks of vincristine, actinomycin-D and doxorubicin based on tumour stage at diagnosis (Fig. 1) [3]. For the comparison of the histopathological WT variants and DWI, additional inclusion criteria for the use of the cutting guide were the availability of an MR study within 10 days after the completion of neoadjuvant chemotherapy (Fig. 1). Paediatric patients with a non-WT were included in the study for general clinical characteristics but were not further analysed concerning the discriminative value of MRI in their differentiation from WTs given their rarity and consequent low numbers (Fig. 1).

### Patient-specific 3D-printed cutting guide

The protocol for the design of the cutting guide was optimised in a prior feasibility study (Supplementary Fig. 1) [27]. The cutting guide was designed to adhere to the same slice thickness (5 millimetres) and orientation of the MR-DW images for the resulting macroscopic slices (M.F., J.N.B.). Lesions were included if at least 75% of the number of slices on DWI were obtained after slicing of the specimen. Moreover, a dice similarity coefficient (1 = perfect overlap, 0 = no overlap) was calculated for the most representative slice containing neoplastic tissue; an index of at least 0.8 was required for the analysis (Fig. 1) [24, 27–29].

### Magnetic resonance imaging acquisition and analysis

All abdominal MR examinations including DWI were performed in our institution on a 1.5-T scanner (*Ingenia; Philips Medical Systems*) (Table 1) [10, 30]. Children were awake, sedated or under general anaesthesia depending on their ability to cooperate, according to the standard of care procedures. Hyoscine butylbromide *(Buscopan; Sanofi)* at a dose of 0.4 mg/kg body weight to reduce peristaltic artifacts (maximum of 5 or 10 mg, depending on age) and Gadobutrol (*Gadovist; Bayer B.V.*) at a dose of 0.1 mL/kg body weight were administered.

**Table 1:** Average scan parameters of the scanned sequences at a 1.5 T magnetic resonance scanner

Parameters	T1W pre-/post-contrast	T2W	DWI
Pulse sequence	2-D ultrafast spoiled GRE with fat suppression	Multivane/TSE with fat suppression	2-D single-shot spin echo (ep) with spectral fat saturation
Slice orientation	Transversal	Transversal/Coronal	Transversal
Repetition time (ms)	5.5	2666	2607
Echo time (ms)	2.7	67	73
Slice thickness (mm)	3	4	5
Echo train length	60	27	35
Acquisition matrix	232/0/0/233	500/0/0/135	88/0/0/70
*B*-values^a^	*NA*	*NA*	0, 25, 50, 100, 150, 200, 250, 500, 800, 1000

*T1W* T1-weighted imaging, *T2W* T2-weighted imaging, *DWI* diffusion-weighted imaging, *ms* milliseconds, *mm* millimetres, *GRE* gradient echo, *TSE* turbo spin echo, *ep* echoplanar

^a^ All patients but three were scanned with 10 *B*-values; 1 patient was scanned with 0, 20, 50, 100, 150, 1000, 1 patient was scanned with 0, 100, 1000, and 1 patient was scanned with 50,800

A paediatric radiologist (ASL) blinded for the histopathological diagnosis of the neoplasm reported the MR examinations as part of clinical care, capturing data in a case report form (CRF) validated through an interobserver agreement study, allowing for only one rater [31]. A (pseudo)capsule was defined as having a low signal intensity at T2-weighted (T2W) imaging. For renal capsule invasion, a focal disruption of the capsule needed to be seen, while discontinuation of the tumour capsule should not automatically indicate extension of the tumour into the peritoneum and/or infiltrative growth pattern. Increased vascularity was defined as a subjective increased number of vessels, often with flow void on T2W imaging. Finally, the intensity of the tumour on T1W and T2W imaging was always considered in comparison to the (contralateral) renal cortex.

### Histopathological review

**Fig. 2:**
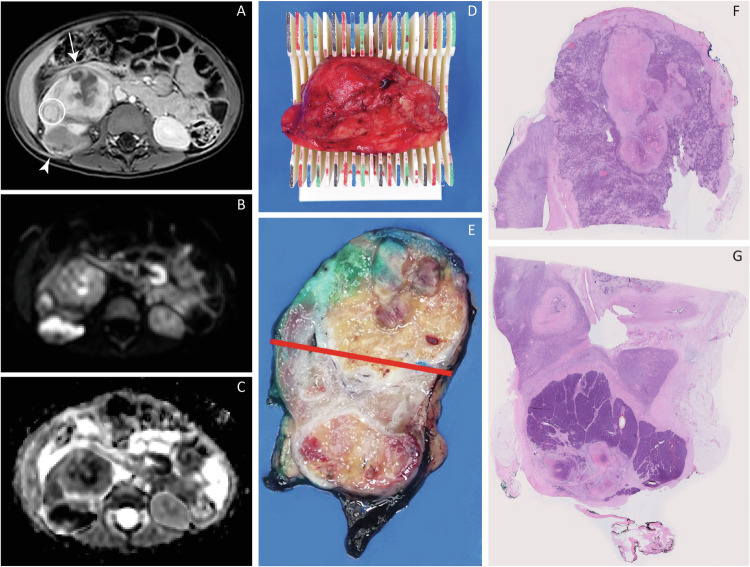
Example of the correlation of imaging and histopathology on a microscopic slide level through the patient-specific 3D-printed cutting guide. Right-sided Wilms’ tumour with multiple lesions in a 27-month-old girl. After neoadjuvant chemotherapy, the contrast-enhanced T1-weighted scan (**A**) showed a ventral lesion with a volume of 41.4 cm^3^ (arrow), a dorsal lesion measuring 10.5 cm^3^ (arrowhead), and a small lateral lesion measuring 0.8 cm^3^ (circle). The largest ventral lesion showed heterogeneous enhancement due to some suspected haemorrhagic components (**A**) and a varying intensity on the B1000- (**B**) and ADC sequence (**C**). The dorsal lesion enhanced more homogeneously, with a high intensity on the B1000- (**B**) and low intensity on the ADC sequence (**C**), indicating high diffusion restriction. The lateral lesion was quite homogeneous with a hypointense capsule (**A**), also showing varying diffusion restrictions (**B**, **C**). A patient-specific 3D-printed cutting guide (**D**) was designed based on the pre-operative MR (**A**–**C**), to slice the specimen in macroscopic slices after nephrectomy in the same orientation and slice thickness as the ADC-scan after nephrectomy (**D**, **E**). All macroscopic slices of the specimen could be directly compared to the pre-operative scan (**A**–**C**, **E**), and selected slices were included in wholemount microscopic histopathological ‘mega’ slides for analysis (**F**, **G**). The ventral lesion was diagnosed as an epithelial WT, the dorsal lesion as a blastemal WT, and the lateral lesion was diagnosed as a hyperplastic perilobar nephrogenic rest (**F**, **G**)

The mass was sliced from cranial to caudal after nephrectomy, following the UMBRELLA protocol. The cutting guide was used in case of a total nephrectomy, in eligible specimens. Macroscopic slices of the specimen were selected for inclusion in microscopic slides according to standard of care [7, 27]. The percentage of chemotherapy-induced changes and of stromal, epithelial and blastemal components were recorded on a whole-tumour level for diagnostic purposes according to UMBRELLA definitions by the local and national review pathologist (M.A.V., R.R.K.). If this was eligible for direct comparison with DW images after slicing in the cutting guide, the macroscopic slices were included in microscopic ‘mega’ slides for separate assessment, following the same UMBRELLA definitions (Figs. 1 and  2) [7].

### Apparent diffusion coefficient measurements

Pseudonymised MR scans of lesions eligible for inclusion with the patient-specific 3D-printed cutting guide were transferred to DICOM software and uploaded in *Horos Project*, and ADC maps were calculated using all available *B*-values (Table 1 and Fig. 1). Freehand regions of interest (ROIs) of the lesions were carefully drawn by the lead investigator (J.N.B.) on the ADC sequence at diagnosis and after neoadjuvant chemotherapy, guided by the T1W contrast-enhanced- and T2W images. Lesions were not assessed if their volume was < 3 cm^3^, resulting in less reliable measurements, and peripheral ROIs were excluded if the surface of the adjacent more central slice on imaging concerned > 50% of the concerning ROI to reduce the partial volume effect. For the WT lesions eligible for direct correlation after slicing with the cutting guide, additional ROIs were drawn in selected ADC slices to correspond with the microscopic ‘mega’ slides (Figs. 1 and  2).

Lesions with predominant cystic or haemorrhagic components were not assessed, based on the estimated lack of reliability of the ADC measurement (Fig. 1) [32, 33]. Furthermore, given the potentially distorted measurements of ADC values in cystic, haemorrhagic and/or necrotic components, ADC histograms on whole-tumour- and slide-level were obtained before and after subtraction of non-enhancing components. The erector spinae muscle on the T1W subtracted sequence was used as a threshold for this purpose [21, 22, 34, 35]. This method previously reported interobserver reliability; hence it was considered sufficient to have one reader, and difficult cases were resolved by a second experienced rater (ASL) [34]. The 25th percentile and median ADC values were obtained, based on promising results in previous studies [18, 19, 21, 22].

### Statistical analysis

The distribution of variables was assessed through visual confirmation and the Shapiro-Wilk test, considering 0.9 to represent normal distribution. For analysis the whole-tumour lesions, ADC values and neoplastic volumes of histologically confirmed WT subtypes were compared using the Mann-Whitney *U-*test, focussing on the difference among stromal, epithelial and blastemal variants. For analysis on a microscopic slide level, the difference of ADC values among these subtypes was assessed using a mixed model (Fig. 1). In case of significant differences, diagnostic performance was analysed through receiver operating curves with an area under the curve (AUC), aiming for the optimal cut-off value exploring sensitivity and specificity. Also, associations between ADC values and percentages of histological WT components were analysed on the microscopic slide level, using Spearman’s rank correlation coefficient (*ρ*) [36]. Based on results in previous studies, a sample size of 32 patients with different proportions of histopathological variants would be sufficient for accurate results (*α* of 0.05; *β* of 0.8) [21, 37]. Taking into consideration the mixed model regression analysis and the correlated analysis on the microscopic slide level through the cutting guide, a 2-year inclusion period was considered conservative, considering the expected number of 30 admitted patients per year on a national basis. Results were deemed significant in the case of *p* < 0.05. Statistical analyses were performed in SPSS *version 27.0* and STATA *version 13*.

## Results

### Patient characteristics

A total of 54 patients (57% males, 43% females) with 59 lesions were included with a median age of 3.0 years (range 0–17.6 years) (Fig. 1 and Table 2). Three of these were diagnosed with non-anaplastic WT after suspicion of a cystic nephroma leading to upfront nephrectomy and were excluded from the analysis. Eight children (15%) presented with bilateral lesions, of which four were affected solely by NRs/nephroblastomatosis. Eight patients presented with a non-WT (Table 2).

**Table 2:** Characteristics of included patients (*n* = 54)

Patient characteristics of all included patients (*n* = 54)	*n*	%
Median age, range *(years)*	3.0 (0 months–17.7 years)	
Gender *(female)*	23	43
Bilateral lesions at presentation	8	15
Metastatic disease	14^a^	26
Lungs	13	
Liver	1	
Bone marrow (with spinal cord compression)	1	
Treatment^b^		
AV 4 weeks	21	38
AVD 6 weeks	16	29
Upfront nephrectomy	11	20
Other^c^	7	13
**Characteristics of all included lesions (*n*** = **59)**^d^	***n***	**%**
Diagnosis		
Wilms’ tumour (with nephrogenic rest (s))	47^e^ *(30)*	80 *(51%)*
Congenital mesoblastic nephroma^f^	4	7
Renal cell carcinoma	2	3
Cystic nephroma	1	2
Nephrogenic rest/Nephroblastomatosis	4	7
Neuroblastoma	1	2
**Characteristics of all included Wilms’ tumour lesions (*n*** = **44)**	***n***	**%**
Median age, range *(years)*	3.6 (4 months–14.8 years)	
Gender *(female)*	23	52
Surgery		
Total nephrectomy	40	91
Nephron-sparing surgery	4	9
Histopathological diagnosis		
Stromal WT	7	16
Epithelial WT	1	2
Blastemal WT	2	5
Mixed WT	12	27
Regressive WT	13	30
Completely necrotic WT	1	2
Diffuse anaplastic WT	8	18

^a^ All patients with metastatic disease were Wilms’ tumour patients, except for one renal cell carcinoma case with pulmonary metastases

^b^ One patient was treated separately for two lesions, resulting in 55 treatment regimens

^c^ Deviations from the protocol were mainly due to vincristine-toxicity, lack of response, or extension of treatment aiming for nephron-sparing surgery

^d^ Of the 54 included patients, three patients presented with two different lesions, whereas two patients underwent separate surgeries which were individually included, resulting in 59 lesions. To avoid ambiguity, hereafter numbers represent lesions

^e^ Three patients (median age 10 months, range 1–21 months) underwent an upfront nephrectomy because of a suspected cystic nephroma/cystic partially differentiated nephroblastoma, resulting in a ‘non-anaplastic WT’. These patients were excluded from whole-tumour analysis given their cystic nature and lack of neoadjuvant chemotherapy

^f^ Two patients were diagnosed with cellular type congenital mesoblastic nephroma (CMN), one patient with classic type CMN, and one patient with mixed type CMN

Patients with a WT (*n* = 39) accounted for a total of 44 WT-lesions and were included for analysis of whole-tumour MR characteristics at the time of diagnosis and after neoadjuvant chemotherapy (Fig. 1 and Table 2). Seven out of 44 lesions (16%) were stromal WT. Eight (18%) lesions were diagnosed as diffuse anaplastic, and only 1 (2%) and 2 (5%) lesions were diagnosed as epithelial and blastemal variants, respectively. The majority (30%) were regressive WTs (Table 2).

### Whole-tumour characteristics of Wilms’ tumours

On baseline MR, WT lesions eligible for whole-tumour analysis of MR characteristics (*n* = 44) predominantly presented as well-defined lobulated masses with a pseudo capsule (98%); cystic components were observed in 73% of the lesions, as well as haemorrhage and necrosis. Both at diagnosis and after neoadjuvant chemotherapy, lesions were predominantly T2W hyperintense and T1W hypointense (Fig. 1 and Table 3).

**Table 3:** MR features of the whole Wilms’ tumours (*n* = 44 lesions)

MRI-characteristics	Diagnostic MRI		Neoadjuvant MRI after pre-operative chemotherapy	
	*n*	%	*n*	%
Median size *(cm*^*3*^*, range)*	549 (3–1957)		185 (1–2090)	
Stromal type WT (*n* = 7)	677 (71–1291)		637 (23–1026)	
Epithelial type WT (*n* = 1)	557		41	
Blastemal type WT (*n* = 2)	50 (17–83)		6 (2–11)	
Mixed type WT (*n* = 12)	468 (9–1957)		103 (6–2090)	
Regressive type WT (*n* = 13)	672 (268–1345)		171 (6–855)	
Completely necrotic (*n* = 1)	171		22	
Diffuse anaplastic WT (*n* = 8)	549 (3–1957)		185 (1–2090)	
Location of the lesion				
Central	14	32	11	25
Peripheral	15	34	22	50
Indistinguishable	15	34	11	25
Regional lymph nodes	4	9	2	5
Shape of the lesion				
Round	18	40	17	39
Lobulated	24	55	23	52
Irregular	2	5	4	9
Tumour margins				
Well-defined	42	95	36	82
Ill-defined	2	5	8	18
Pseudo capsule	43	98	39	89
Renal capsule invasion/rupture	21	48	19	43
Infiltrative growth pattern	3	7	3	7
Venous invasion (tumour thrombus)	5	11	3	7
Haemorrhage/Necrosis	31	70	33	75.0
Cysts (Septations)	32 (3)	73 (9)	30 (9)	68 (30)
Fatty tissue	2	5	1	2
Subcapsular fluid	7	16	3	7
Increased vascularity	1	2	0	0.0
T2-weighted imaging intensity^a^				
Hypointense	1	2	6	14
Hyperintense	34	77	27	61
Isointense	5	11	5	11
Varying intensity	4	9	6	14
T2-weighted imaging appearance				
Homogeneous	18	40	5	11
Heterogeneous	26	59	39	89
T1-weighted imaging intensity^a^				
Hypointense	39	89	29	66
Hyperintense	3	7	3	7
Isointense	0	0.0	9	21
Varying intensity	2	5	3	7
T1-weighted imaging appearance				
Homogeneous	18	41	9	21
Heterogeneous	26	59	35	80
T1-weighted contrast enhancement				
Homogeneous	15	34	13	30
Heterogeneous	29^b^	66	31^c^	70
Median ADC-value^d^ *(×10*^*-3*^ *mm*^*2*^*/s, range)*				
Stromal type WT (*n* = 7)	1.026 (0.747–1.221)		1.437 (1.140–1.504)	
Epithelial type WT (*n* = 1)	0.841		1.015	
Blastemal type WT (*n* = 2)	0.600 (0.516–0.683)		0.628 (0.596–0.660)	
Mixed type WT (*n* = 12)	0.777 (0.630–1.170)		1.172 (0.825–1.748)	
Regressive type WT (*n* = 12)	0.862 (0.645–1.179)		1.299 (1.092–1.748)	
Diffuse anaplastic WT (*n* = 7)	0.998 (0.654–1.318)		1.442 (0.570–1.744)	

*MRI* magnetic resonance imaging, *ADC* apparent diffusion coefficient, *WT* Wilms’ tumour

^a^ Hypointensity, hyperintensity and isointensity were assessed compared to the normal (healthy) renal parenchyma

^b^ Two tumours showed band-like areas of later or non-enhancement

^c^ One tumour showed band-like areas of later or non-enhancement, one lesion showed only enhancement of the septae and two tumours showed solely peripheral capsule enhancement

^d^ Whole-tumour ADC-values based on the histology after neoadjuvant chemotherapy and nephrectomy for *n* = 41 eligible lesions (Fig. 1)

WT lesions had a median size of 549 cm^3^ (range 3–1957 cm^3^) at diagnosis and 185 cm^3^ (range 1–2090 cm^3^) after neoadjuvant chemotherapy (Table 3). Stromal WT demonstrated the lowest response, with a median decrease of 48 cm^3^ (range decrease of 562 cm^3^—an increase of 333 cm^3^ (*p* = 0.035). Regressive neoplasms showed the best response to chemotherapy with a median decrease of 335 cm^3^ (range 82–1067 cm^3^) (Table 3).

In total, 41/44 (93%) WT lesions were eligible for whole-tumour ADC measurements at diagnosis and after neoadjuvant chemotherapy (Fig. 1). The lesions classified as blastemal WT (*n* = 2) showed the lowest whole-tumour ADC measurements at the time of diagnosis and especially after completion of neoadjuvant chemotherapy (median of 0.600 * 10^−3^ and 0.628 * 10^−3^ mm^2^/s, respectively). Stromal WTs (*n* = 7) showed a median increase of 0.393 * 10^−3^ mm^2^/s (range 0.283–0.537 * 10^−3^ mm^2^/s), whereas this was significantly less in the epithelial and blastemal masses (*n* = 3) (0.080 * 10^−3^ mm^2^/s, range −0.023 to 0.174 * 10^−3^ mm^2^/s) (*p* = 0.017). Regressive WTs exhibited a wide ADC range after surgery with low ADC values at diagnosis, with a large increase in values after neoadjuvant chemotherapy (Table 3).

### Direct correlation of histopathology and diffusion-weighted imaging on a microscopic slide level

Thirty-two WT lesions were eligible for correlation of histology and ADC values on a microscopic slide level through the cutting guide, resulting in the analysis of 240 microscopic ‘mega’ slides (Figs. 1 and  2). The median time required to design the cutting guide was 55 min (range 28 min–16 h). The median time for 3D printing was 27 h (range 15 h–3 days), with a median cost of €24 (range €11–58).

**Fig. 3:**
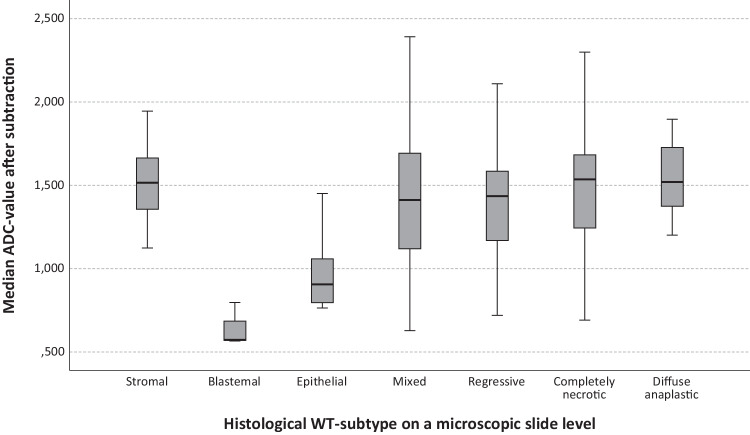
Box-and-whisker plots show the overall subtracted median ADC values after neoadjuvant chemotherapy for Wilms’ tumours on a microscopic slide level (*n* = 240 slides) after direct correlation through the patient-specific 3D-printed cutting guide. The median (middle line), quartile (top and bottom of the box) and extreme values (whiskers) for median ADC values after neoadjuvant chemotherapy, based on the direct correlation of histopathology and DWI on a microscopic ‘mega’ slide level in specimens eligible for inclusion after use of the patient-specific 3D-printed cutting guide (*n* = 240) for stromal (*n* = 84), blastemal (*n* = 3), epithelial (*n* = 18), mixed (*n* = 48), regressive (*n* = 58), completely necrotic (*n* = 14) and diffuse anaplastic (*n* = 15) areas of the lesions. ADC, apparent diffusion coefficient (*10^-3^ mm^2^/s); WT, Wilms’ tumour; DWI, diffusion-weighted imaging

Median ADC value of microscopic ‘mega’ slides was 1.516 * 10^−3^ mm^2^/s (range 1.124–1.945 * 10^−3^ mm^2^/s) for stromal areas (*n* = 84), compared to 0.906 * 10^−3^ mm^2^/s (range 0.764–1.451 * 10^−3^ mm^2^/s) and 0.573 * 10^−3^ mm^2^/s (range 0.566–0.797 * 10^−3^ mm^2^/s) for epithelial (*n* = 18) and blastemal (*n* = 3) foci of the WT, respectively (Fig. 3). Median ADC for stromal areas was significantly different from epithelial and blastemal areas combined (*p* < 0.001). Analysis of the optimal cut-off value for stromal WT resulted in a sensitivity of 95.2% (95% confidence interval 87.6–98.4%) and specificity of 90.5% (95% confidence interval 68.2–98.3%) for a median ADC value of 1.195 * 10^−3^ mm^2^/s, with an AUC of 0.961 (95% CI 0.912–1.000) (Supplementary Fig. 2).

Finally, the proportion of stromal, blastemal and epithelial components after analysis on microscopic ‘mega’ slide level showed a fair linear relationship with 25th percentile and median ADC values after neoadjuvant chemotherapy. Stromal areas showed the strongest linear relationship with 25th percentile ADC values (*ρ* = 0.490, *p* < 0.001) (Supplementary Fig. 3A). Furthermore, the analysis demonstrated the strongest inverse linear relationship for median ADC with the proportion of blastemal components and 25th percentile ADC with the proportion of epithelial components (*ρ* = −0.274, *p* = 0.005, and *ρ* = −0.445, *p* < 0.001, respectively) (Supplementary Fig. 3B, C).

### Nephrogenic rests

In total, 30/44 (64%) WT lesions included NR; 4 were solitary hyperplastic perilobar and homogeneously T1-hypointense and T2-hyperintense, measuring from 0.6 to 11 cm^3^, and three of them showed a capsule (Table 2). Baseline ADC values (*n* = 3) ranged from 0.538–0.751 * 10^−3^ mm^2^/s, whereas one NR was too small for measurements.

## Discussion

In this prospective national study, we showed that MR-DWI can be used to discriminate the histological WT subtypes. Our results are in concordance with previous studies that found an association among ADC values, histopathological findings, and architectural changes related to neoadjuvant chemotherapy [12, 18–23, 25, 30, 38]. Nevertheless, previous results were predominantly based on whole-tumour analyses of WT. We included the correlation between histology and DWI on a microscopic slide level through a patient-specific 3D-printed cutting guide, providing highly accurate radiological data and a highly discriminative cut-off ADC value for stromal WT [27]. Previous studies demonstrated that the latter is less aggressive than the blastemal or diffuse anaplastic variants after neoadjuvant chemotherapy, which are associated with poor outcomes. Nonetheless, identification of stromal type WT is clinically relevant in the light of additional neoadjuvant chemotherapy after standard neoadjuvant treatment aiming for nephron-sparing surgery in bilateral cases, given the often-seen limited shrinkage [8, 19, 37, 39, 40]. The increasing ability of DWI as a non-invasive diagnostic tool to help discriminate paediatric renal neoplasms at an early stage, combining both morphological and semi-quantitative data of the lesions, is especially important for patients treated in the SIOP-RTSG setting [8, 17, 19, 22, 30, 39].

At baseline, WT usually presents as a large heterogeneous encapsulated mass with haemorrhagic, necrotic, and cystic components with a tumour thrombus, displacing the adjacent structures [9, 10, 38, 41, 42]. The high percentage of haemorrhagic, cystic, and necrotic components in this study is, therefore, in line with previous results, as is the percentage of venous invasion (11%) [6, 10, 30, 43–47]. As expected, 98% of WT patients (98%) showed a (pseudo) capsule at diagnosis; the renal parenchymal invasion was seen in almost half of the patients [48]. Also in our cohort, WTs appeared typically T1W hypointense and T2W hyperintense, heterogeneously vascularised and with a high-cellular solid component [20, 41, 45–47, 49].

Previous studies concluded chemotherapy generally reduces the tumour volume, which is also one of the main aims of neoadjuvant treatment in SIOP-RTSG protocols [3, 20, 50, 51]. However, the traditional response assessment based on the change in size of imaging has become less important over the past decades and has even been discouraged as a sole marker [45, 52, 53]. As confirmed in this large prospective study, stromal WT has been associated with a tendency to differentiate into more mature stromal or mesenchymal components following the neoadjuvant chemotherapy; whereas, the volume might remain unaltered or even increase [23, 37, 50, 51]. Furthermore, blastemal type WT is the most chemosensitive variant, often markedly shrinking. Nevertheless, shrunken lesions may still contain predominantly malignant cells, such as chemo-resistant blastemal cells, associated with poorer event-free and overall survival [8, 20, 21, 38, 50–52, 54].

Stromal lesions showed relatively high ADC values of the whole mass at diagnosis, with an increase after neoadjuvant chemotherapy, suggested to be related to the differentiation of the tissue [18, 20–22]. Furthermore, we found that median and 25th percentile ADC values on a whole-tumour level on the baseline and response assessment MR-DWI scans were lower in lesions with remaining blastema after chemotherapy [18–22]. Likewise, the rather low ADC values at baseline seen in pathologically proven regressive lesions, together with their previously discussed decrease in volume, could suggest a chemosensitive blastemal nature [8, 10, 20, 21, 38].

In this context, Hötker et al advocated for further analysis of the ADC histogram distribution, including median, percentile, kurtosis, and skewness of the ADC values for better risk stratification [19]. However, this study focused on whole-tumour ADC values, disregarding underlying heterogeneous histopathology and leading to less accurate correlations [21, 55–59]. Littooij et al proposed that a direct correlation of single-slice ADC measurements with a matched histopathology slice could make the evaluation more specific [21]. This retrospective visual correlation led to a varying degree of comparability due to differences in slice thickness and orientation [21, 60]. The cutting guide, as demonstrated in an earlier feasibility study, enabled comparison of slices concerning orientation and thickness in this study. As expected, the only issues were caused by large cystic neoplasms [27, 61]. Analyses of directly correlated data on a microscopic slide level resulted in a significant difference in median ADC values of stromal areas compared to epithelial and blastemal areas of the WT. Although this is in line with previous retrospective results, this study has additionally provided a highly significant cut-off value with high sensitivity and specificity, suggesting clinical applicability [22]. Since measurements were based on enhancing components, representing viable neoplastic tissue, these ADC values may refer to WT variants regardless of the assessment before or after the neoadjuvant chemotherapy.

Mixed WT lesions were difficult to evaluate due to the wide range of ADC values, which is related to the heterogeneous composition of the subtype. Furthermore, despite the exclusion of haemorrhagic and necrotic components, regressive WT also showed wide ranges of ADC values. Necrosis is related to low cellularity; whereas haemorrhagic areas can mimic high-cellular components in case of coagulation, explaining these findings [32, 33]. Therefore, these foci are usually both identified T1W sequences with and without gadolinium administration [32]. Finally, early recognition of diffuse anaplastic WT may have important clinical implications. Despite a lack of discriminative features, ADC values in this study were rather high; this seems in line with the report by Hötker et al, showing a significantly increased ADC value of the 75th percentile for diffuse anaplastic lesions, which was suggested to be due to a greater amount of regressive changes [18].

This study has several limitations. First, patients were treated with neoadjuvant chemotherapy, resulting in an expected rather high number of regressive specimina and a low number of epithelial and blastemal ones. Hence, the ADC values of the microscopic slides of the latter were combined as more aggressive subtypes in the discriminative analysis from stromal type WTs. Second, although the correlation of histopathology and DWI slices after the use of the cutting guide resulted in more specific and larger amounts of data, this only allowed for correlation after neoadjuvant chemotherapy, while the response to it could only be assessed on a whole-tumour level. Nonetheless, the exclusion of non-enhancing components from the measurements resulted in ADC values of viable neoplastic tissue, which may be clinically applicable for discrimination of subtypes regardless of the assessment before or after drug administration. Third, analysis of MR characteristics was only performed by one rater, based on tested interobserver variability of the CRF and measurement of whole-tumour ADC values [31, 34]. This novel approach on slice and slide level might be subject to a somewhat broader variability, whereas also variability in ADC values originating from different MR equipment needs to be taken into consideration concerning validation for international use, despite the specified SIOP-RTSG 2016 UMBRELLA MR-DWI acquisition protocol [21, 62, 63]. Nonetheless, despite the existing variability related to imaging features, we feel the methods used and choice for a single rater have not caused limitations regarding the reliability of the results. Finally, the rarity of non-WTs limited the ability to focus on their discrimination from WTs, especially in the light of the identification of features that should prompt consideration of biopsy. Ongoing inclusion as well as international collaboration may lead to potential analyses of the discriminative value of MR-DWI for the differentiation of WTs and non-WTs, while previously identified general solid tumour characteristics on other MR-sequences should also be taken into consideration [17, 31, 64, 65]. NRs which were separately identifiable and measurable on histopathology and on MR were also scarce, limiting strong conclusions concerning their differentiation from small WTs. Nonetheless, ongoing inclusion and analysis of non-WTs and NRs using correlation through the cutting guide might allow future discriminative analyses.

In conclusion, the correlation between histopathology and MR-DWI after slicing in the innovative cutting guide resulted in a highly significant discrimination of stromal type WT based on ADC-value, together with limited shrinkage and a considerable increase in ADC-values after chemotherapy. Especially in bilateral cases, potentially eligible for nephron-sparing surgery, stromal tumours might not benefit from additional pre-operative chemotherapy given the potentially limited decrease in size, whereas in general the early recognition of high-risk histopathology might facilitate more personalised treatment already from diagnosis on. Yet, reliable differentiation between epithelial and blastemal lesions remains difficult due to the rarity of these histological types after neoadjuvant chemotherapy and a similar marked diffusion restriction. Collaborative international efforts, analysing, for instance, treatment-naïve WTs, might enable replication, validation, and inclusion of a higher number of epithelial and blastemal-type tumours.

## Supplementary information

ELECTRONIC SUPPLEMENTARY MATERIAL

## Abbreviations

ADC: Apparent diffusion coefficient
AUC: Area under the curve
DWI: Diffusion-weighted imaging
MR-DWI: Magnetic resonance imaging
NR: Nephrogenic rest
ROI: Region of interest
SIOP-RTSG: International Society of Paediatric Oncology-Renal Tumour Study Group
T1W: T1-weighted
T2W: T2-weighted
WT: Wilms’ tumour
