# Supplementary material for: Direct correlation of MR-DWI and histopathology of Wilms’ tumours through a patient-specific 3D-printed cutting guide
Source: Eur Radiol. 2024 Aug 8;35(2):652–63. doi: 10.1007/s00330-024-10959-2 (PMC11782413; doi:10.1007/s00330-024-10959-2)
Supplement: Supplementary file 1 — ELECTRONIC SUPPLEMENTARY MATERIAL [file 330_2024_10959_MOESM1_ESM.docx]

**Direct correlation of MR-DWI and histopathology of Wilms’ tumours through a patient-specific 3D-printed cutting guide: a national prospective study**

**SUPPLEMENTARY MATERIALS**

CONTENT

**Supplementary Figure 1.** The use of the patient-specific 3D-printed cutting guide in the clinical workflow for pediatric renal tumors

**Supplementary Figure 2.** Differentiation of stromal Wilms’ tumour (*n*=84 slides) compared to epithelial- and blastemal Wilms’ tumour (*n*=21 slides) based on the median ADC value after neoadjuvant chemotherapy on a microscopic slide level after direct correlation through the patient-specific 3D-printed cutting guide, shown in (A) a box-and-whisker plot and (B) an ROC curve analysis

**Supplementary Figure 3.** Correlation of the relation between proportion of Wilms’ tumour components (%) and ADC values (*10^-3^ mm^2^/s) after neoadjuvant chemotherapy on a microscopic slide level through the patient-specific 3D-rpinted cutting guide for (A) the 25^th^ percentile ADC values and proportion of stromal areas, (B) the median ADC values and proportion of blastemal areas) and (C) the 25^th^ percentile ADC value and proportion of epithelial areas

**Supplementary Figure 1.** The use of the patient-specific 3D-printed cutting guide in the clinical workflow for pediatric renal tumors [1] *(used with permission)*


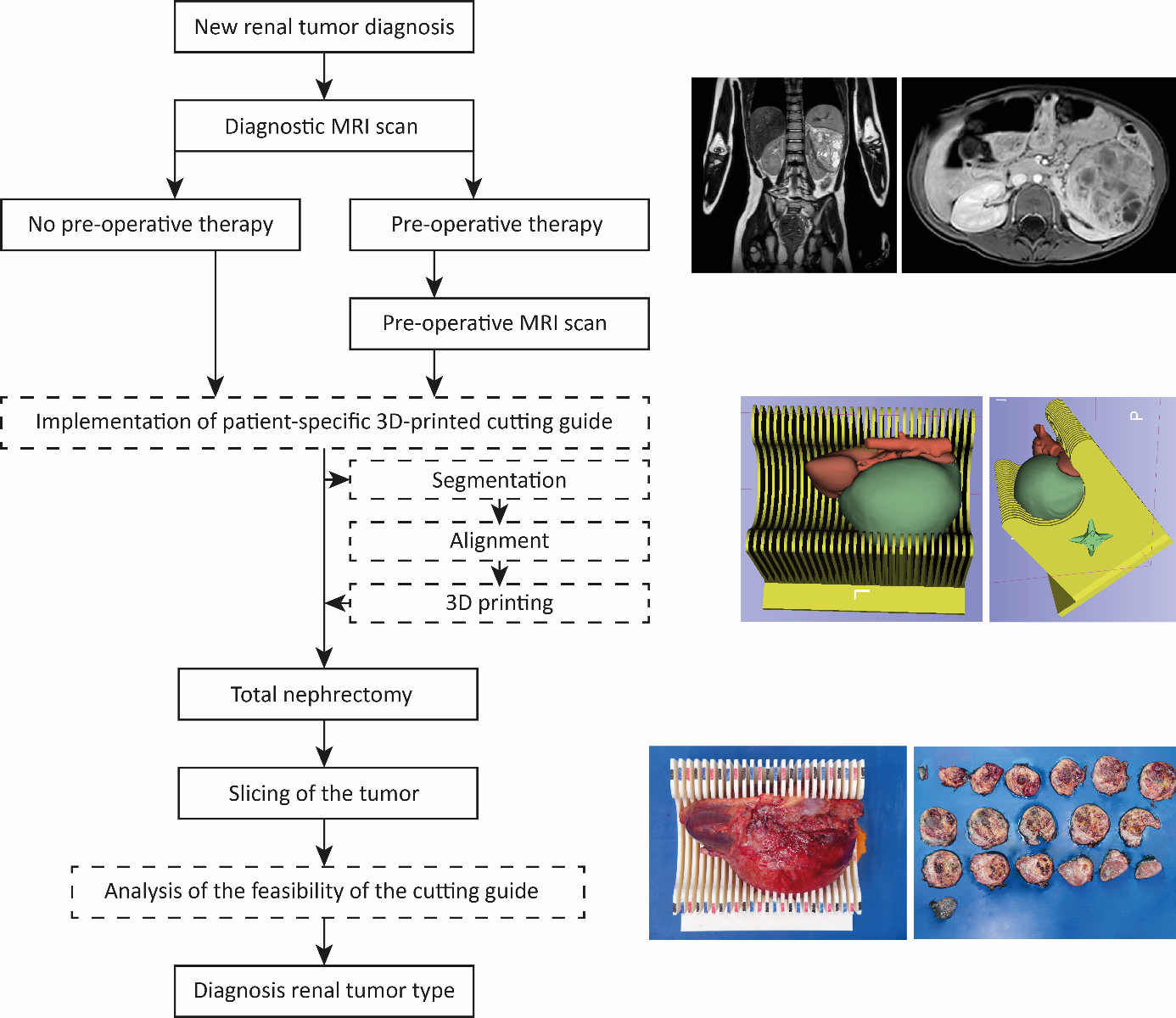


**Supplementary Figure 2.** Differentiation of stromal Wilms’ tumour (*n*=84 slides) compared to epithelial- and blastemal Wilms’ tumour (*n*=21 slides) based on the median ADC value after neoadjuvant chemotherapy on a microscopic slide level after direct correlation through the patient-specific 3D-printed cutting guide, shown in (A) a box-and-whisker plot and (B) an ROC curve analysis

(A) (B)


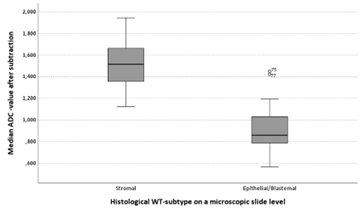

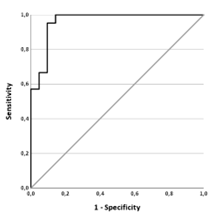


The box-and-whisker plot (A) shows the median *(middle line)*, quartiles *(top and bottom of the box)*, extreme values *(whiskers)* and outliers *(circles)* for the overall median ADC value after neoadjuvant chemotherapy for stromal type WT (*n*=84) and epithelial- and blastemal type WT (*n*=21), based on the direct correlation of histopathology and DWI on a microscopic ‘mega’ slide level in specimens eligible for inclusion after use of the patient-specific 3D-printed cutting guide. The ROC curve (B) analysis for differentiation of stromal type WT from epithelial- and blastemal type WT combined shows an AUC of 0.961 (95%CI 0.912-1.000), with an optimal cut-off value of 1.195*10^-3^ mm^2^/s resulting in a sensitivity of 95.2% (95%CI 87.6-98.4%) and specificity of 90.5% (95%CI 68.2-98.3%).

ADC = apparent diffusion coefficient (*10^-3^ mm^2^/s); WT = Wilms’ tumour; DWI = diffusion weighted imaging.

**Supplementary Figure 3.** Correlation of the relation between proportion of Wilms’ tumour components (%) and ADC values (*10^-3^ mm^2^/s) after neoadjuvant chemotherapy on a microscopic slide level through the patient-specific 3D-rpinted cutting guide for (A) the 25^th^ percentile ADC values and proportion of stromal areas, (B) the median ADC values and proportion of blastemal areas) and (C) the 25^th^ percentile ADC value and proportion of epithelial areas

(A)


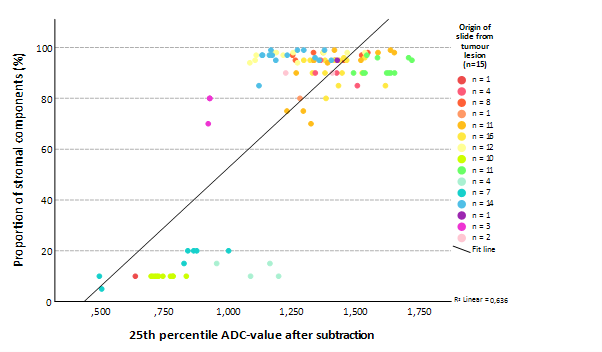


(B)


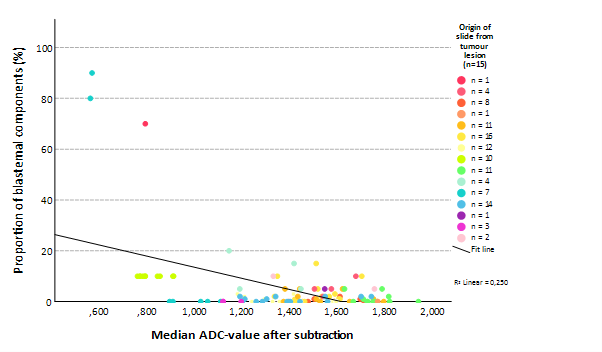


(C)


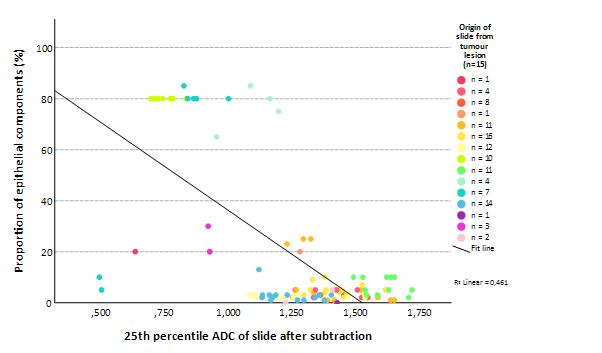


Proportion (%) of stromal, blastemal and epithelial components in stromal (*n*=84), blastemal (*n*=3) and epithelial (*n*=18) microscopic ‘mega’ slides after neoadjuvant chemotherapy with indication of tumour lesions origin (*n*=15, indication by colours) based on the direct correlation of histopathology and DWI on a microscopic slide level in specimens eligible for inclusion after use of the patient-specific 3D-printed cutting guide, resulting in a fair linear relationship for 25^th^ percentile ADC value of stromal components (ρ=0.490, p<0.001), median ADC value of blastemal components (ρ=-0.274, p=0.005) and 25^th^ percentile ADC value of epithelial components (ρ=-0.445, p<0.001).

ADC = apparent diffusion coefficient (*10^-3^ mm^2^/s); DWI = diffusion weighted imaging.

**REFERENCES**

1 van der Beek JN, Fitski M, de Krijger RR et al (2023) Direct correlation of MRI with histopathology in pediatric renal tumors through the use of a patient-specific 3-D-printed cutting guide: a feasibility study. Pediatr Radiol 53:235-243
